# Supplementary material for: Capture at the ER-mitochondrial contacts licenses IP3 receptors to stimulate local Ca2+ transfer and oxidative metabolism
Source: Nat Commun. 2022 Nov 9;13:6779. doi: 10.1038/s41467-022-34365-8 (PMC9646835; doi:10.1038/s41467-022-34365-8)
Supplement: Supplementary file 1 — Supplementary Information [file 41467_2022_34365_MOESM1_ESM.pdf]

Capture at the ER-Mitochondrial Contacts Licenses IP_3 receptors to Stimulate Local Ca^{2+} Transfer and Oxidative Metabolism

Katona et al.

Fig. S1

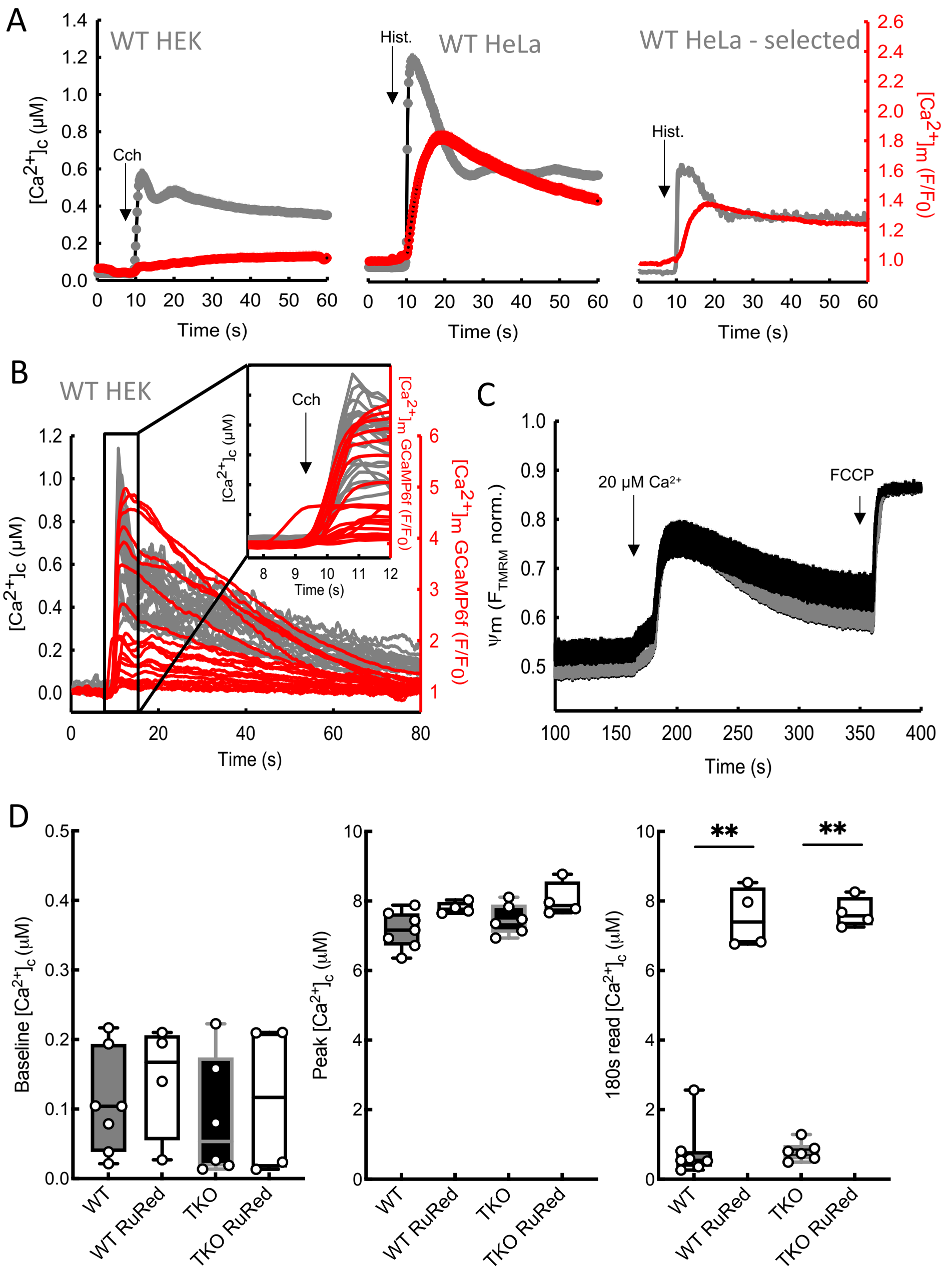

Fig. S1 WT HEK cells showed weak ER-mitochondrial Ca^{2+} coupling, but similar mitochondrial Ca^{2+} uptake to IP3R deficient cells

(A) Average time courses showing agonist-induced (Carbachol or Histamine) $[Ca^{2+}]_c$ and $[Ca^{2+}]_m$ changes in WT HEK (left), WT HeLa (middle) and selected WT HeLa to match WT HEK responses (right). (Datapoints represent averages with ±S.E.M.) (B) Representative traces showing Cch-induced changes in $[Ca^{2+}]_c$ (gray) and corresponding changes in matrix targeted GCaMP6f (red) fluorescence with kinetics similar to the $[Ca^{2+}]_c$ response in HEK cells, suggesting cytosolic localization of GCaMP6f (n=21). (C) Time courses of TMRM fluorescence in suspensions of permeabilized WT (gray) and TKO HEK (black) cells presenting changes in ΔΨm after Ca^{2+} pulse and FCCP. (Datapoints represent averages with ±S.E.M.) (D) Box plots showing the baseline $[Ca^{2+}]_c$, peak $[Ca^{2+}]_c$ and $[Ca^{2+}]_c$ after 180s with (WT – dark gray, TKO – black) and without (empty) RuRed pretreatment (WT, TKO n=7, WT+RuRed, TKO+RuRed n=4; ** < 0.001, One-way ANOVA, Holm-Sidak method, box plots indicate median, 25th and 75th percentile (box), and 5th and 95th percentile (whiskers) and all datapoints). Source data are provided for each panel Source Data file.

Fig. S2

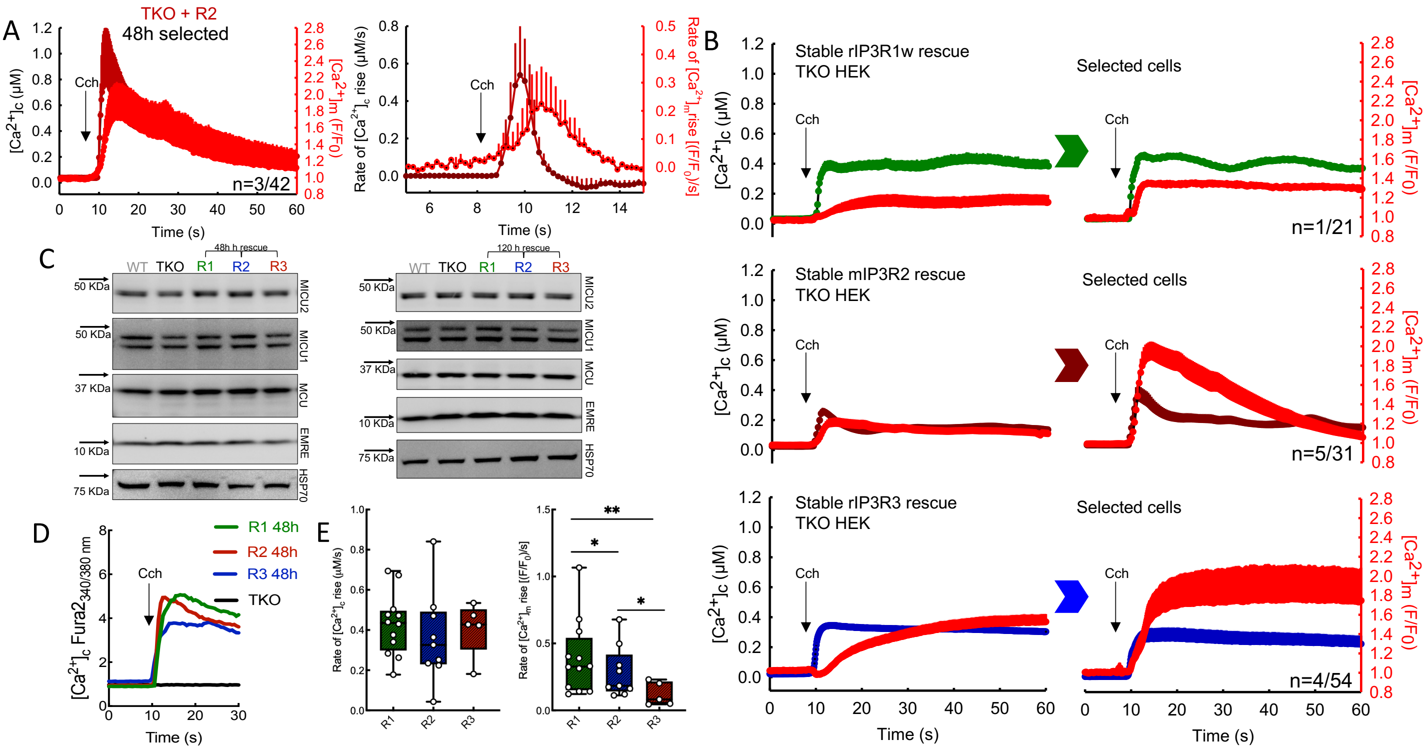

Fig. S2 Long term overexpression of IP3Rs did not alter the expression of the mitochondrial Ca^{2+} uniporter complex, while promoted ER-mitochondrial Ca^{2+} communication

(A) Average time courses of the changes in $[Ca^{2+}]_c$ (maroon) and $[Ca^{2+}]_m$ (red) in selected 48 h R2 acute rescue cells with less than 3s cyto-mito coupling time (left). Mean traces of the rate of Ca2+ rise in the cytosol and the mitochondrial matrix of the selected cells (right). (Datapoints represent averages with ±S.E.M.) (B) Average traces of the $[Ca^{2+}]_c$ and $[Ca^{2+}]_m$ agonist responses in all cells (left) and a selected subpopulation of well coupled IP3R stable rescue cells (right). (Datapoints represent averages with ±S.E.M.) (C) Immunoblots showing the protein levels of MCU, MICU1, MICU2 and EMRE in WT, TKO HEK and IP3R1-3 acute 48h (left) and 120h (right) rescue TKO HEK lysates. (D) Average [Ca2+]c time courses confirming IP3R-rescue in cells processed for immunoblots in panel C. (E) Calculated maximum rates of $[Ca^{2+}]_c$ and $[Ca^{2+}]_m$ change in selected cells for each 120 h IP3R rescue condition. (* $p<0.05$, ** $p<0.001$, One-way ANOVA, Normality Test (Shapiro-Wilk), All Pairwise Multiple Comparison Procedures (Holm-Sidak method), box plots indicate median, 25th and 75th percentile (box), and 5th and 95th percentile (whiskers) and all datapoints. Source data are provided for each panel as a Source Data file.

FigS3

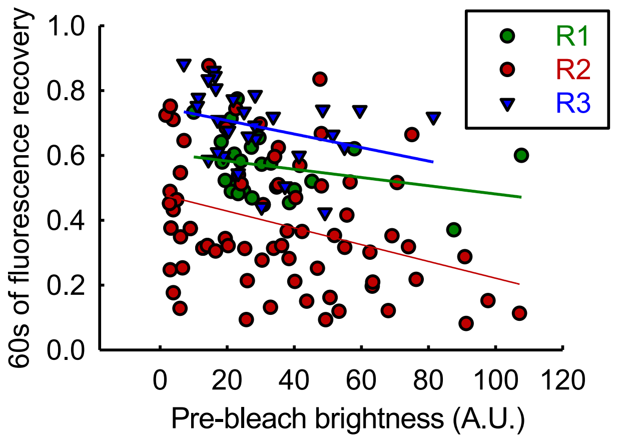

Fig. S3 Pre-bleach brightness had no effect on fluorescence recovery

Extent of fluorescence recovery after photobleaching by IP3R isoform plotted as a function of the pre-bleach brightness under identical imaging conditions (IP3R1 – green, IP3R2 – red, IP3R3 – blue). Source data are provided as a Source Data file.

FigS4

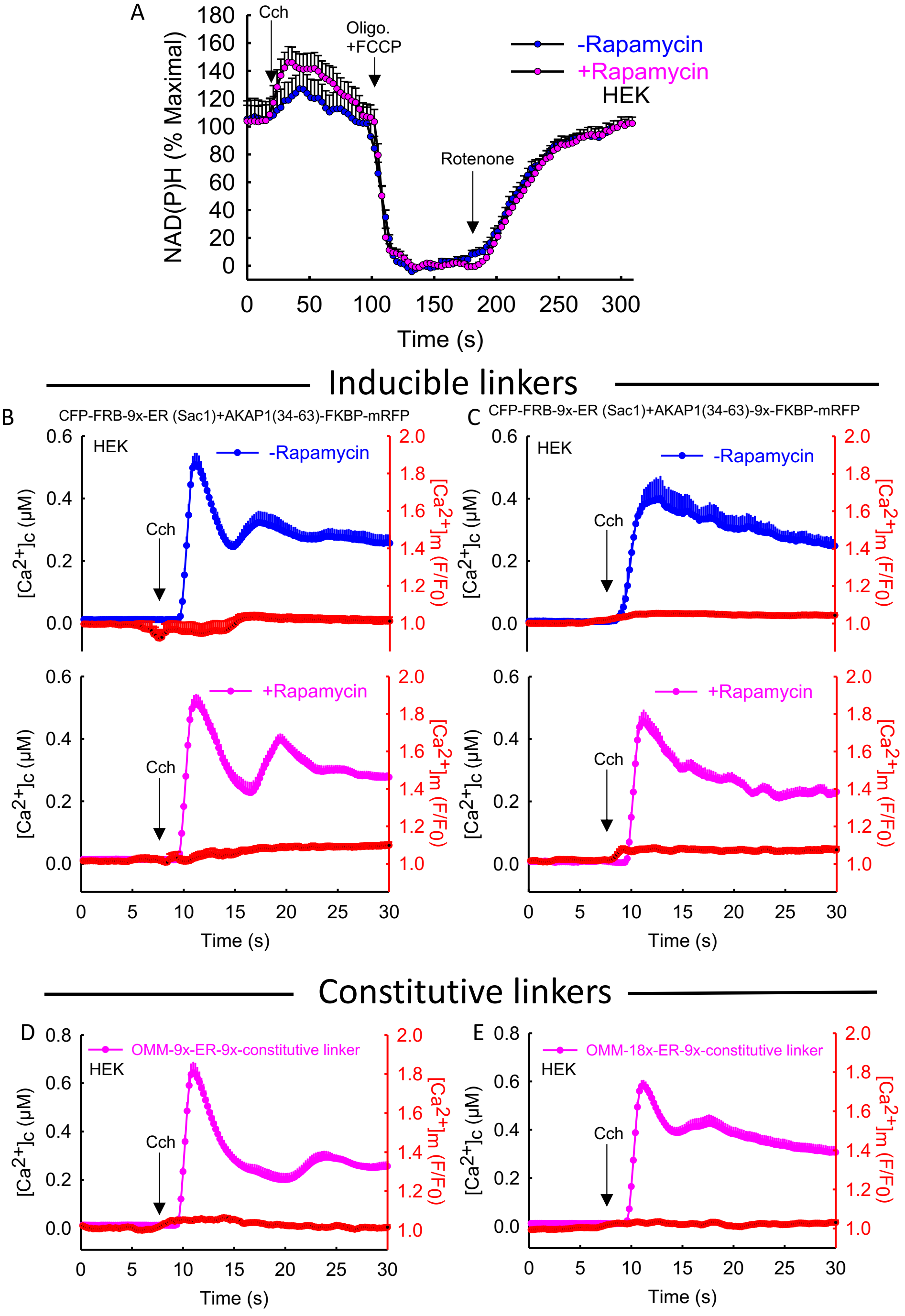

Fig. S4 IP3R3 localization to the ERMCs by linkage promoted oxidative metabolism, while increasing ERMCs by linkers had no effect on ER-mitochondrial Ca^{2+} transfer in WT HEK

(A) Average time courses showing agonist (Cch) induced changes in NAD(P)H autofluorescence with and without trapping R3 at the ERMCs by rapamycin-induced linkage (n=21). (B-C) Average Cch induced $[Ca^{2+}]_c$ and corresponding $[Ca^{2+}]_m$ responses in WT HEK cells expressing rapamycin inducible (B) SHORT (1x helix; -Rapa n=11, +Rapa n=17) or (C) LONG (9x helix; -Rapa n=10, +Rapa n=12) ER-FRB and OMM-FKBP linker pair without (blue) and with (pink) rapamycin treatment. (D-E) Average $[Ca^{2+}]_c$ and $[Ca^{2+}]_m$ responses in WT HEK cells expressing constitutive (D) LONG (18x helix, n=10) or (E) EXTRA LONG (27x helix, n=14) ER-OMM linker pair expressing cells. (Datapoints represent averages with ±S.E.M. in all graphs.) Source data are provided for each panel as a Source Data file.
