# Supplementary material for: Capture at the ER-mitochondrial contacts licenses IP3 receptors to stimulate local Ca2+ transfer and oxidative metabolism
Source: Nat Commun. 2022 Nov 9;13:6779. doi: 10.1038/s41467-022-34365-8 (PMC9646835; doi:10.1038/s41467-022-34365-8)
Supplement: Supplementary file 3 — Description of Additional Supplementary Files [file 41467_2022_34365_MOESM3_ESM.pdf]

Description of Additional Supplementary Files

File Name: Supplementary Movie 1
Description: Time lapse showing mRFP-FRB-R3 (purple, top left) co-localizing with OMM-FKBPYFP(green, top right) after rapamycin treatment. mRFP-YFP overlay confirming R3 re-localization to the vicinity of OMM (white, bottom right). ER-lumen targeted CFP showing unaltered ER morphology during R3 co-localization with the OMM (cyan, bottom left).
