# Supplementary material for: Capture at the ER-mitochondrial contacts licenses IP3 receptors to stimulate local Ca2+ transfer and oxidative metabolism
Source: Nat Commun. 2022 Nov 9;13:6779. doi: 10.1038/s41467-022-34365-8 (PMC9646835; doi:10.1038/s41467-022-34365-8)
Supplement: Supplementary file 5 — Reporting Summary [file 41467_2022_34365_MOESM5_ESM.pdf]

nature portfolio

Corresponding author(s): Gyorgy Hajnoczky

Last updated by author(s): 2022- 10-13

Reporting Summary

Nature Portfolio wishes to improve the reproducibility of the work that we publish. This form provides structure for consistency and transparency in reporting. For further information on Nature Portfolio policies, see our Editorial Policies and the Editorial Policy Checklist.

Statistics

For all statistical analyses, confirm that the following items are present in the figure legend, table legend, main text, or Methods section.

n/a	Confirmed	
☐	☒	The exact sample size (n) for each experimental group/condition, given as a discrete number and unit of measurement
☐	☒	A statement on whether measurements were taken from distinct samples or whether the same sample was measured repeatedly
☐	☒	The statistical test(s) used AND whether they are one- or two-sided<br>*Only common tests should be described solely by name; describe more complex techniques in the Methods section.*
☒	☐	A description of all covariates tested
☐	☒	A description of any assumptions or corrections, such as tests of normality and adjustment for multiple comparisons
☐	☒	A full description of the statistical parameters including central tendency (e.g. means) or other basic estimates (e.g. regression coefficient) AND variation (e.g. standard deviation) or associated estimates of uncertainty (e.g. confidence intervals)
☐	☒	For null hypothesis testing, the test statistic (e.g. F, t, r) with confidence intervals, effect sizes, degrees of freedom and P value noted<br>*Give P values as exact values whenever suitable.*
☒	☐	For Bayesian analysis, information on the choice of priors and Markov chain Monte Carlo settings
☒	☐	For hierarchical and complex designs, identification of the appropriate level for tests and full reporting of outcomes
☒	☐	Estimates of effect sizes (e.g. Cohen's d, Pearson's r), indicating how they were calculated

Our web collection on statistics for biologists contains articles on many of the points above.

Software and code

Policy information about availability of computer code

Data collection	PTI Felix GX Photon Technology International, Inc Version 4.2; Zen Carl Zeiss Microscopy Gmbh Version 2.3; Vutara SRX Bruker Version 5.23, 6.0, 6.01; ERMito Analysis (ImageJ Plugin) in house (Open source) http://sites.imagej.net/MitoCare/; Excel Microsoft 2010 and 2016
Data analysis	Sigmaplot Systat Software, Inc. Version 12.5; ImageJ National Institutes of Health, USA Fiji (IJv1.52); Python Open source (Anaconda, Inc.) Version 2.7; ER Mito Analysis (ImageJ plugin) In-house (Open source) http://sites.imagej.net/MitoCare/; Excel Microsoft 2010 and 2016

For manuscripts utilizing custom algorithms or software that are central to the research but not yet described in published literature, software must be made available to editors and reviewers. We strongly encourage code deposition in a community repository (e.g. GitHub). See the Nature Portfolio guidelines for submitting code & software for further information.

Data

Policy information about availability of data

All manuscripts must include a data availability statement. This statement should provide the following information, where applicable:

- Accession codes, unique identifiers, or web links for publicly available datasets
- A description of any restrictions on data availability
- For clinical datasets or third party data, please ensure that the statement adheres to our policy

The datasets generated during and/or analyzed during the current study are available from the corresponding author on reasonable request.

Human research participants

Policy information about studies involving human research participants and Sex and Gender in Research.

Reporting on sex and gender	N/A
Population characteristics	N/A
Recruitment	N/A
Ethics oversight	N/A

Note that full information on the approval of the study protocol must also be provided in the manuscript.

Field-specific reporting

Please select the one below that is the best fit for your research. If you are not sure, read the appropriate sections before making your selection.

☒ Life sciences ☐ Behavioural & social sciences ☐ Ecological, evolutionary & environmental sciences

For a reference copy of the document with all sections, see nature.com/documents/nr-reporting-summary-flat.pdf

Life sciences study design

All studies must disclose on these points even when the disclosure is negative.

Sample size	We did not use any statistical method to pre-determine sample sizes. Our goal was to obtain a reasonable sample size during each experiments, depending on the nature of the experiment. in TEM and immunofluorescence imaging, a minimum of 10 cells were analyzed per condition per fixation. FRET and Ca2+ imaging was performed with a large field detector, therefore the number of individual cells were multiples of the performed runs. For these experiments 5-10 independent runs were performed per experimental day.
Data exclusions	No data points were excluded.
Replication	All main figure data was obtained in at least 3 successful independent sets of experiments.
Randomization	Measurements (cytosolic and mitochondrial Ca2+ levels,) performed on different cell lines on the same day were randomized to avoid the detection of possible differences caused by different incubation times by distributing samples through the experimental day (e.g. following transfection or cell plating).
Blinding	Electron micrograghs were analyzed blinded. Other experiments were not performed blinded.

Reporting for specific materials, systems and methods

We require information from authors about some types of materials, experimental systems and methods used in many studies. Here, indicate whether each material, system or method listed is relevant to your study. If you are not sure if a list item applies to your research, read the appropriate section before selecting a response.

Materials & experimental systems

n/a	Involved in the study
☐	☒ Antibodies
☐	☒ Eukaryotic cell lines
☒	☐ Palaeontology and archaeology
☒	☐ Animals and other organisms
☒	☐ Clinical data
☒	☐ Dual use research of concern

Methods

n/a	Involved in the study
☒	☐ ChIP-seq
☒	☐ Flow cytometry
☒	☐ MRI-based neuroimaging

Antibodies

Antibodies used

FLAG Sigma F1804-200UG 088K6018
IP3R3 BD Transduction laboratories 610312 51627
IRDye 800CW Donkey Anti-Rabbit LiCor 926-32213 C60712-05
AF647 Rabbit Anti-Mouse Thermo Fisher A-21239 1774710
CF568 Goat Anti-Rabbit IgG Biotium 20103-1 16C0422
AF647 Goat anti-Rabbit IgG Thermo Fisher A-21244 1818084
Calnexin ENZO ADI-SPA-860
mtHSP70 (JG1) Invitrogen MA3-028
IP3R1 (CT1) custom made, Joseph SK, Samanta S. 1993, PMID: 8384211
IP3R2 custom made, Pocono Rabbit Farms and Laboratories
IgG Nanogold Nanoprobes, 2001-0.5ml 06D222
anti-HA Abcam ab911
AF594 anti-rabbit IgG Molecular Probes A-11037

Validation

Commercially available antibodies were validated by the manufacturers in different molecular biological applications. References for applications are provided by the manufacturers. α-IP3R1 was validated before (Joseph SK, Samanta S. 1993, PMID: 8384211) and for this study, comparing IP3R1 expressing wild-type, knockout and rescued KO cell lines with confocal microscopy, as well as with western blot, both shown in the manuscript. α-IP3R2 was validated with western blot, comparing IP3R2 expressing wild-type, knockout and rescued KO cell lines, shown in the manuscript.

Eukaryotic cell lines

Policy information about cell lines and Sex and Gender in Research

Cell line source(s): WT and IP3 deficient TKO HEK293 cells were provided by David I. Yule (Univ Rochester https://www.kerafast.com/productgroup/703/ip3r-expressing-hek-293-cell-lines

Authentication: cells were validated by stimulation with Carbachol and by Western blot

Mycoplasma contamination: All cell lines were tested negative for mycoplasma

Commonly misidentified lines (See ICLAC register): No commonly misudentified cells were studied

Palaeontology and Archaeology

Specimen provenance: *Provide provenance information for specimens and describe permits that were obtained for the work (including the name of the issuing authority, the date of issue, and any identifying information). Permits should encompass collection and, where applicable, export.*

Specimen deposition: *Indicate where the specimens have been deposited to permit free access by other researchers.*

Dating methods: *If new dates are provided, describe how they were obtained (e.g. collection, storage, sample pretreatment and measurement), where they were obtained (i.e. lab name), the calibration program and the protocol for quality assurance OR state that no new dates are provided.*

☐ Tick this box to confirm that the raw and calibrated dates are available in the paper or in Supplementary Information.

Ethics oversight: *Identify the organization(s) that approved or provided guidance on the study protocol, OR state that no ethical approval or guidance was required and explain why not.*

Note that full information on the approval of the study protocol must also be provided in the manuscript.

Animals and other research organisms

Policy information about studies involving animals; ARRIVE guidelines recommended for reporting animal research, and Sex and Gender in Research

Laboratory animals	*For laboratory animals, report species, strain and age OR state that the study did not involve laboratory animals.*
Wild animals	*Provide details on animals observed in or captured in the field; report species and age where possible. Describe how animals were caught and transported and what happened to captive animals after the study (if killed, explain why and describe method; if released, say where and when) OR state that the study did not involve wild animals.*
Reporting on sex	*Indicate if findings apply to only one sex; describe whether sex was considered in study design, methods used for assigning sex. Provide data disaggregated for sex where this information has been collected in the source data as appropriate; provide overall numbers in this Reporting Summary. Please state if this information has not been collected. Report sex-based analyses where performed, justify reasons for lack of sex-based analysis.*
Field-collected samples	*For laboratory work with field-collected samples, describe all relevant parameters such as housing, maintenance, temperature, photoperiod and end-of-experiment protocol OR state that the study did not involve samples collected from the field.*
Ethics oversight	*Identify the organization(s) that approved or provided guidance on the study protocol, OR state that no ethical approval or guidance was required and explain why not.*

Note that full information on the approval of the study protocol must also be provided in the manuscript.

Clinical data

Policy information about clinical studies

All manuscripts should comply with the ICMJE guidelines for publication of clinical research and a completed CONSORT checklist must be included with all submissions.

Clinical trial registration	*Provide the trial registration number from ClinicalTrials.gov or an equivalent agency.*
Study protocol	*Note where the full trial protocol can be accessed OR if not available, explain why.*
Data collection	*Describe the settings and locales of data collection, noting the time periods of recruitment and data collection.*
Outcomes	*Describe how you pre-defined primary and secondary outcome measures and how you assessed these measures.*

Dual use research of concern

Policy information about dual use research of concern

Hazards

Could the accidental, deliberate or reckless misuse of agents or technologies generated in the work, or the application of information presented in the manuscript, pose a threat to:

No	Yes	
☒	☐	Public health
☒	☐	National security
☒	☐	Crops and/or livestock
☒	☐	Ecosystems
☒	☐	Any other significant area

Experiments of concern

Does the work involve any of these experiments of concern:

No	Yes	
☒	☐	Demonstrate how to render a vaccine ineffective
☒	☐	Confer resistance to therapeutically useful antibiotics or antiviral agents
☒	☐	Enhance the virulence of a pathogen or render a nonpathogen virulent
☒	☐	Increase transmissibility of a pathogen
☒	☐	Alter the host range of a pathogen
☒	☐	Enable evasion of diagnostic/detection modalities
☒	☐	Enable the weaponization of a biological agent or toxin
☒	☐	Any other potentially harmful combination of experiments and agents
